# LymAnalyzer: a tool for comprehensive analysis of next generation sequencing data of T cell receptors and immunoglobulins

**DOI:** 10.1093/nar/gkv1016

**Published:** 2015-10-07

**Authors:** Yaxuan Yu, Rhodri Ceredig, Cathal Seoighe

**Affiliations:** 1 School of Mathematics, Statistics and Applied Mathematics, National University of Ireland Galway, University Road, Galway, Ireland; 2 Biosciences, National University of Ireland Galway, University Road, Dangan, Galway, Ireland

## Abstract

The adaptive immune system includes populations of B and T cells capable of binding foreign epitopes via antigen specific receptors, called immunoglobulin (IG) for B cells and the T cell receptor (TCR) for T cells. In order to provide protection from a wide range of pathogens, these cells display highly diverse repertoires of IGs and TCRs. This is achieved through combinatorial rearrangement of multiple gene segments in addition, for B cells, to somatic hypermutation. Deep sequencing technologies have revolutionized analysis of the diversity of these repertoires; however, accurate TCR/IG diversity profiling requires specialist bioinformatics tools. Here we present LymAnalzyer, a software package that significantly improves the completeness and accuracy of TCR/IG profiling from deep sequence data and includes procedures to identify novel alleles of gene segments. On real and simulated data sets LymAnalyzer produces highly accurate and complete results. Although, to date we have applied it to TCR/IG data from human and mouse, it can be applied to data from any species for which an appropriate database of reference genes is available. Implemented in Java, it includes both a command line version and a graphical user interface and is freely available at https://sourceforge.net/projects/lymanalyzer/.

## INTRODUCTION

T cell receptors (TCRs) and immunoglobulins (IGs) recognize diverse arrays of foreign antigens and play important roles in the adaptive immune response. The diversity of TCRs and IGs is achieved by V(D)J recombination (for both TCRs and IGs) and somatic hypermutation (for IGs). V(D)J recombination is a stochastic process of rearrangement of variable (V), joining (J) and diversity (D, for the TCR beta chain and IG heavy chain only) gene segments during the early stages of T and B cell maturation. Somatic hypermutation is the T cell-dependent process through which IGs undergo extremely high rates of somatic mutation during the proliferation of B cells in germinal centres. As a consequence of this hypermutation process, B cells are selected for their expression of higher affinity IGs, a process called affinity maturation. The complementarity determining region 3 (CDR3) which includes part of the V, all of the D and some of the J gene segments is the most variable region of TCR/IG sequences and plays the major role in binding specificity. In man, theoretical estimates of the number of distinct TCR and IG generated by this mechanism are around 10^10^ (1). The analysis of CDR3 diversity within individuals reveals insights into the mechanisms of adaptive immunity as well as clinically relevant information about the state of the immune system in individual patients (2). Therefore, robust bioinformatics pipelines for comprehensive analysis of TCR/IG diversity are required.

Compared to the Sanger sequencing technology, next generation sequencing (NGS) technology provides information at much higher resolution about the DNA sequences of TCR and IG, allowing more complete analysis of lymphocyte repertoires. This gives us an opportunity to gain a better understanding of adaptive immunity. Typically, the main objectives are to identify the VDJ genes, extract the CDR3 region and estimate the diversity of the lymphocyte repertoire. Existing software packages are available for VDJ identification and CDR3 extraction. IgBlast (3) and IMGT/High-V-Quest (4) are both web-based tools for TCR/IG sequence analysis that make use of dynamic programming sequence alignment algorithms. These tools include user-friendly graphical user interfaces (GUIs), and they are fast and robust enough for the analysis of small numbers of TCR/IG sequences. iHMMune-align (5) uses a hidden Markov model to align IG sequences. However, for high throughput sequencing data sets, these three tools are no longer suitable due to the limited numbers of sequences they can process (no more than 150 000 reads), as all of these tools were developed for sequence data generated by traditional sequencing technologies. More recently, Decombinator (6) and MiTCR (7) were developed specifically for the analysis of NGS data from TCRs (neither tool currently allows the analysis of IG sequences). MiXCR (8) is the most recently developed tool for the analysis of TCR/IG data. However, we demonstrate here that techniques used to achieve the speed required for the analysis of NGS data by these tools result in reduced accuracy in VDJ gene assignment and an incomplete profile of TCR diversity. Here we present LymAnalyzer, a software package for the comprehensive and accurate analysis of TCR/IG NGS data. The alignment step in LymAnalyzer, which is based on a fast-tag-searching algorithm, results in rapid identification of VDJ gene segments, with significantly improved accuracy and completeness compared to existing tools applied to TCR data. In addition, LymAnalyzer can be applied to IG sequences, includes an integrated single nucleotide polymorphism (SNP) calling algorithm that identifies novel alleles of the VDJ gene segments and produces lineage mutation trees to represent the affinity maturation process of the IGs.

## MATERIALS AND METHODS

### The workflow of LymAnalyzer

**Figure 1.**
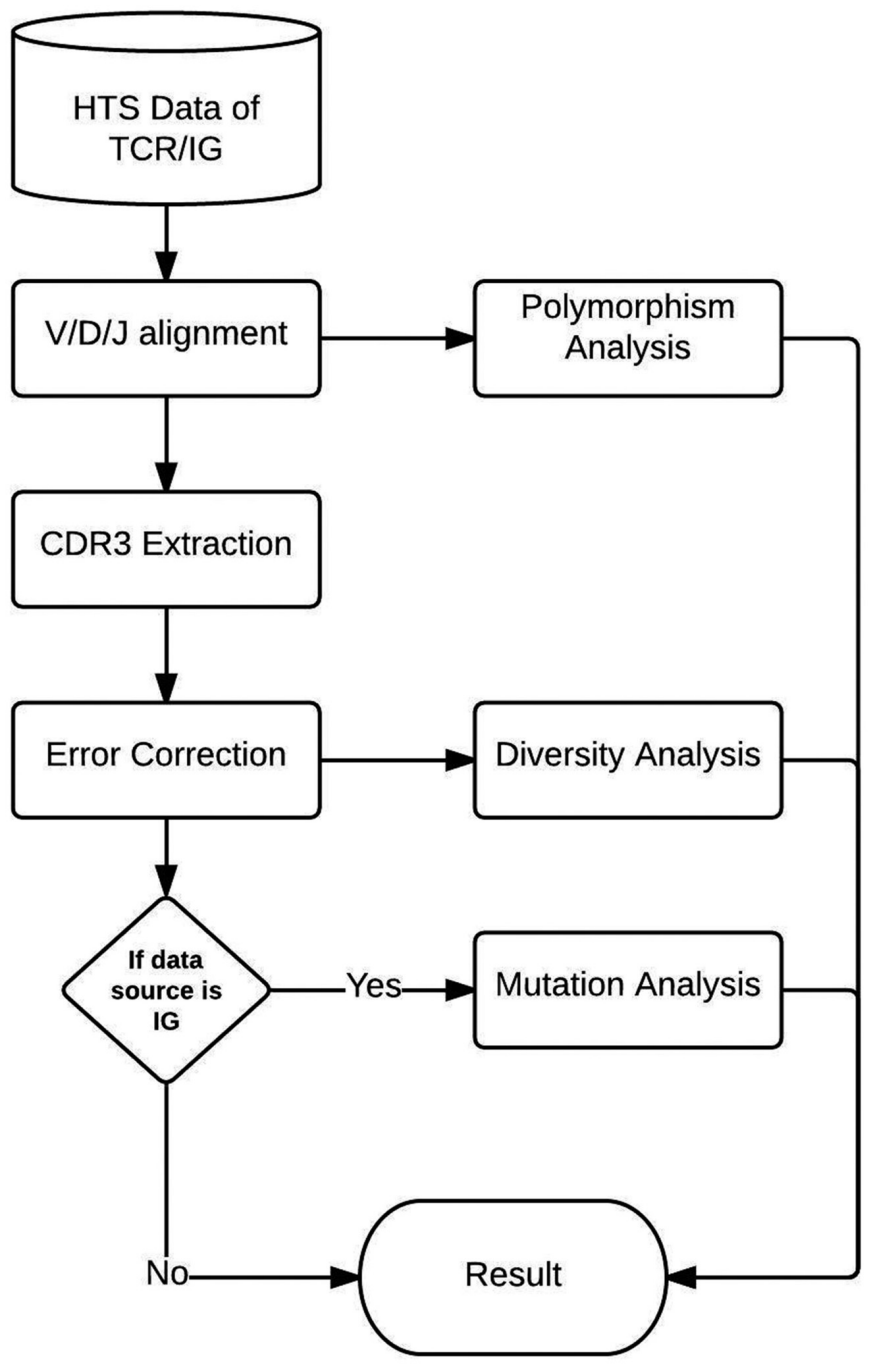
The stepwise workflow of LymAnalyzer.

LymAnalyzer consists of four functional components: VDJ gene alignment, CDR3 extraction, polymorphism analysis and lineage mutation tree construction (Figure 1).

TCR/IG Diversity analysis is the first process in the pipeline. This process includes three steps, the first of which is V/D/J alignment. For each input sequence, we use a fast-tag-searching algorithm, described in detail below, to determine the reference V, D and J genes from which this input sequence is derived. Each input sequence is aligned against all sequences in the International Immunogenetics Database (IMGT) (9) and the best matching V, D and J sequences are selected. In the second step we extract the CDR3 region from the sequence. The CDR3 region of TCR/IG begins with the last cysteine of the V segment and ends with the conserved motif [FW] GXG (X represents any amino acid) in the J segment. The conserved motif in the J segment is straightforward to identify in the input sequence because 12 nucleotides are sufficient to ensure unique occurrence within the TCR/IG sequences. But for the cysteine motif in the V segment, there may be two cysteines both located towards the end of the sequence. In our pipeline, for each of the input sequences, we first find the location of the last cysteine of the corresponding reference V gene we obtained from the alignment step. We then map this location back to the input sequence to determine the position of the last cysteine on the input sequence. This allows us to determine the sequence of the CDR3 region. The third and final step involves classification of the CDR3 sequences. After we obtain the CDR3s from previous step, we classify the input sequences. CDR3 sequences are clustered into clonotypes and the number of clonotypes and the number of sequences per clonotype are calculated. This process is discussed in detail below.

Users can also choose to perform polymorphism analysis to identify novel SNPs that do not correspond to alleles contained in the IMGT database. Each potential SNP, as well as the observed frequencies of the alternative alleles, is included in the result report. For IGs, by default LymAnalyzer will also create lineage trees that describe the stepwise somatic hypermutation of IG sequences in the germinal centre (10).

### NGS data for TCRs/IGs

We obtained TCR/IG sequence data from the NCBI Sequence Read Archive (SRA) to test the performance of LymAnalyzer. The experimental data consisted of two data sets. The TCR sequence data (SRA index: PRJNA229070) used here is from Putintseva *et al*. (11). It consists of nine samples; the number of reads in each sample ranges from 4 202 419 to 13 872 805. The reads are all from the beta chain of TCR covering part of V, all the D and part of the J region (100 bp long). A second data set, consisting of IG sequences, (SRA index: SRP017087) is from Doria-Rose *et al*. (12). This data set contains seven samples, with read counts varying from 271 382 to 23 191 224. Each sequence is 250 bp long and comes from the heavy or light chain of the IG. It contains part of V (all the D for heavy chain) and part of the J region. Putintseva et al. used MiTCR to analyse the TCR data, Doria-Rose et al. exploited their own bioinformatics pipelines, which included BLAST to process the IG data.

### Simulated data set

We used simulation to compare the accuracy of LymAnalyzer and existing tools. Firstly we created a reference gene database: the reference V, D and J gene database used in our simulation pipeline is obtained from the latest version of IG/TCR repertoire of IMGT database. For each of the simulated sequences, we selected the V, D and J gene segments assuming uniform gene usage from the reference gene database. Mismatches were introduced to simulate the combined effects of polymerase chain reaction (PCR) errors, sequencing errors and mutations/polymorphisms in the input sequences, each of which can lead to differences between the input sequences and the corresponding gene segments in the database. Three mismatch levels were used: no mismatches, a low mismatch level and a high mismatch level. For the low mismatch level, there were 0–7 mismatch(es) on the V gene, 0–1 mismatch on the D gene and 0–3 mismatch(es) on the J gene. For the high mismatch level, there were 0–15 mismatch(es) on the V gene, 0–2 mismatch(es) on the D gene and 0–5 mismatch(es) on the J gene. The number and the position of the introduced mismatch(es) in the corresponding gene were both uniformly distributed. After obtaining the ‘mutated’ V, D and J segments, we added 0–6 randomly generated nucleotides to the V-D and D-J junction to simulate nucleotide insertions during VDJ recombination. We generated three data sets, with varying mismatch rates, each consisting of 20 samples. Each of the samples contained 200 000 TCR/IG sequences.

### Fast-tag-search based alignment algorithm

**Figure 2.**
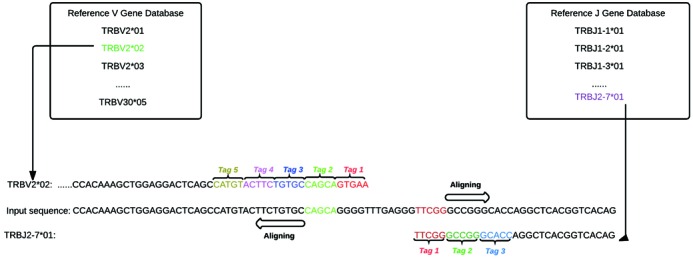
Alignment algorithm: Reference V and J genes are shown in the boxes on the right and left. The 5 detection tags of one of the reference V genes (TRBV2*02) are labelled with different colours within the V gene sequence (left). Tag 1 (red) fails to achieve an exact match with the input sequence but an exact match with tag 2 (green) can be used to extend an alignment in both directions. Similarly exact matching of tag 1 (red) seeds the alignment of J sequence TRBJ2–7*01 (right) to the input.

Due to the large size of NGS data sets fast algorithms are required for sequence alignment. LymAnalyzer uses an alignment algorithm based on fast-tag-searching to map the input sequence to reference V and J segments. We first define a detection tag set that consists of multiple short detection tags from the input string. Iteratively we use detection tags from the tag set to search for perfect matches in the second string and store the indexes that obtain perfect matches. Subsequently we calculate the Hamming distance (the number of positions at which the corresponding symbols are different) of these two strings by extending from each perfect match index (Figure 2). By default, the reference VDJ genes used by LymAnalyzer are derived from the most recent update of the IMGT database; however, users can also choose to import their own reference gene database. For each of the reference genes, we select the last five nucleotides from the 3′ end of the sequence as our first detection tag T_1_. Then we select another five nucleotides, which are located adjacent to the previous detection tag by extending towards the 5′ end. The same operations are repeated until we obtain five detection tags (The number of the tags is an adjustable parameter that can be defined by users; it is five in the default setting) and we get the detection set V as

(1) \begin{equation*} {\rm V} = \{ {\rm T}_1 ,{\rm T}_2 ,{\rm T}_3 ,{\rm T}_4 ,{\rm T}_5 \} \end{equation*}

For any reference J genes, instead of choosing the last five nucleotides of the 3′ end, the algorithm starts from the 5′ end. The same operations as we described for the reference V gene are repeated three times (This is an adjustable parameter that can be defined by users; it is three in the default setting) to get the J set where

(2) \begin{equation*} {\rm J} = \{ {\rm T}_1 ,{\rm T}_2 ,{\rm T}_3 \} \end{equation*}

Furthermore, we locate the indexes that have perfect matches with the first tag, T_1_, for each of the input sequences. However this may not be successful due to mutations and sequencing errors in the matching region; hence, the five detection tags used for the V genes and three detection tags used for the J genes. If the preceding tag fails, we used the subsequent tag to repeat the matching. Once we find perfect matching index(es), we extend in both directions from this index and calculate the matching score, which consists of the number and percentage of matches between the input and reference sequence. If the number of matches passes the minimum threshold (i.e. 90% match and 30 bp matches), we keep the corresponding reference gene in the candidate set. After this process is applied to all of the reference genes, we choose the sequence with the highest percentage of matches from the candidate set. As J genes are shorter than V genes, we use only three detection tags in the J genes and the minimum threshold requires only 20 bp matches. D gene alignment is different from V and J gene alignment because D genes are short (12–16 bp) relative to V and J genes, D genes are quite similar to each other and the D gene is inside the CDR3 region, which is hyper variable. Once we have successfully aligned the V and J genes, we remove the region that is aligned with them. The remaining sequence contains only the D gene. We choose the last three nucleotides from the 3′ end and 5′ end of each reference D gene as our detection tags. Again we use the detection tags to find a perfect match and extend to get a matching score. Subsequently we select the D gene, which has the highest matching score and passes the minimum matching threshold (90% match and 10 bp matches), as our aligned D gene segment.

### CDR3 extraction and classification

Once the reference VDJ genes of each input sequence have been determined, we extract the CDR3 sequence and classify the input sequences by their CDR3. Input sequences are in the same CDR3 class if they are mapped to the same V(D)J genes and have identical CDR3 region nucleotide sequences. CDR3 classification takes place in two stages: We firstly perform preliminary classification based on exact matching of the extracted CDR3 sequence and count the number of each clonotype (CDR3 classes). This results in large numbers of singleton clonetypes and clonetypes that have small numbers of copies. CDR3 sequences with counts below an adjustable portion (default = 0.001%) of the sequences are labelled as ‘minimum sequences’, with the rest of the sequences labelled as ‘core sequences’. For each of the minimum sequences, we calculate its Hamming distance to the core sequences of the same length. If the Hamming distance is less than M steps (M is an adjustable parameter, default = 2), we merge the minimum sequence with the corresponding core sequence. This process is repeated by iterating over the minimum sequences.

### SNP calling

After the input sequence has been aligned with the corresponding reference genes, it is straightforward to locate nucleotides that do not match the reference sequence. These are considered as potential SNPs in the input sequences. In order to avoid treating PCR errors as potential polymorphisms we use two criteria described by Schott et al. (13). The first is that the same gene variant should occur in multiple V(D)J combinations. For instance, when we are searching for V gene SNPs we require the potential non-reference allele on the V gene to associate with more than three different J genes. As we have more V genes than J genes, the minimum number of different V genes required to define a potential SNP on the J gene is five. The second criterion to identify a candidate SNP is that the non-reference allele should occur at a frequency of at least 10% among the sequences of the corresponding gene. This is informed by the assumption that somatic point mutations should occur in fewer than 5% of all the sequences, unless they are within the G/C mutation hot spot, in which case they can reach a frequency of 10% (13).

**Figure 3.**
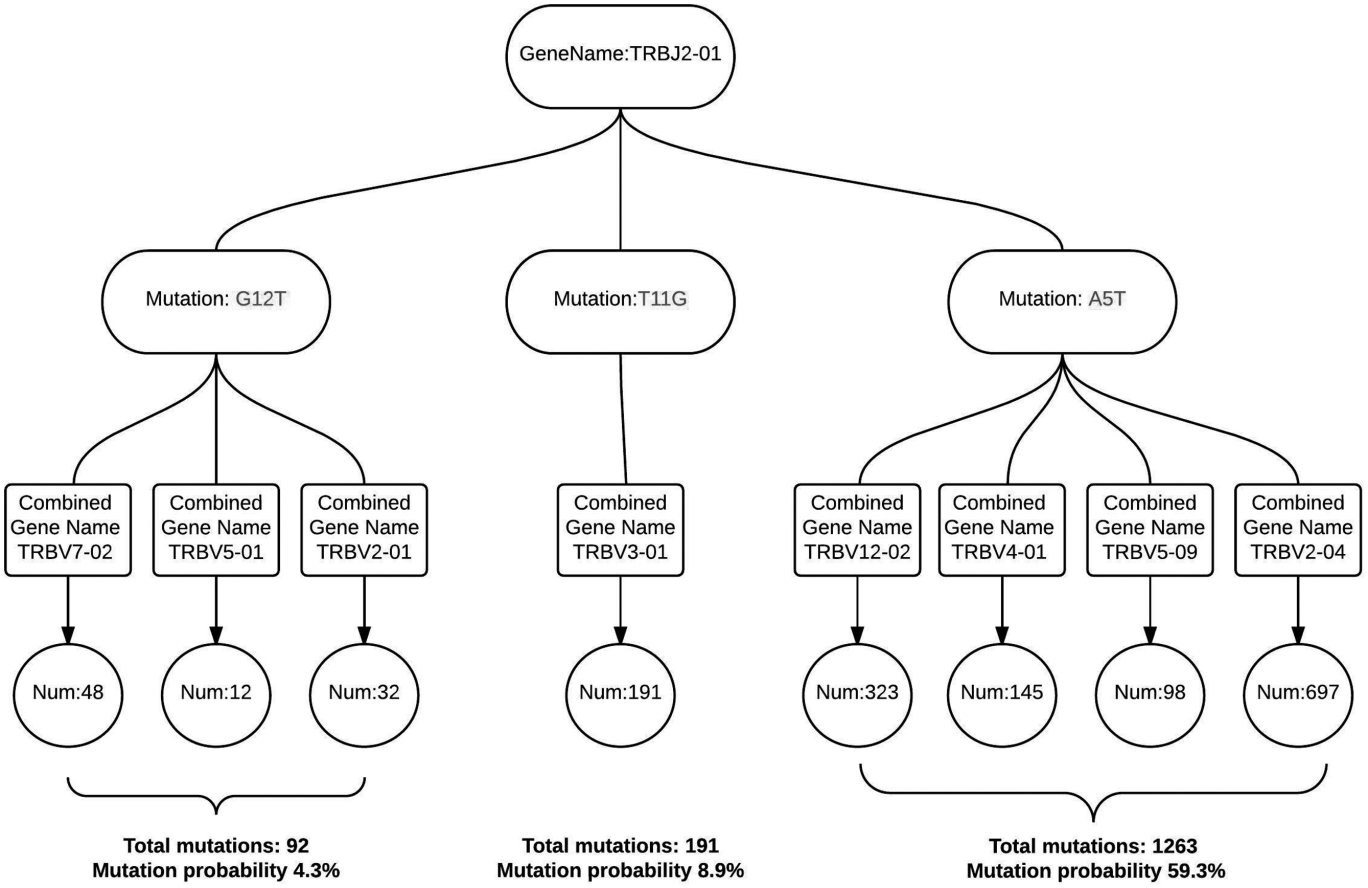
Data structure used in SNP analysis. For each of the reference genes, we have a Hashmap storing the mutation information. For reference J gene TRBJ2–01, three mutations are detected (shown in red). For example, G12T means Glycine at position 12, mutated to Tyrosine. This mutation is found in combination with three different V segments with a combined frequency of 4.3%.

In order to efficiently store and index the potential SNPs, we use a nested Hashmap structure (Figure 3). For each of the mutations found in a given reference gene, we calculate the percentage of mutations and the number of different gene types associated with them. If the two criteria mentioned above are met we store it in the potential SNP data set for downstream analysis.

### Mutation tree construction

LymAnalyzer creates lineage mutation trees for IGs. The lineage mutation tree construction algorithm used in LymAnalyzer is based on the modification of the distance method concept exploited by Barak *et al*. (14). As noted by the authors, this method does not aim to simulate the particular mutation process that occurred. Instead it aims to reveal the minimal steps that could have led to the observed sequences. We firstly define the root sequences, which are those sequences with the original germline configuration. For each of the root sequences, we find the sequences that are within 10 Levenshtein steps (the minimum steps required to change one string to another only using insertions, deletions or substitutions). Each layer of the tree is created according to the distance to the root node.

### Statistical test

We used two approaches to test the statistical significance of differences in proportions of mapped reads. Treating individual sequence reads as the statistical unit, the equality of the proportion of mapped reads (and, in the case of the simulated data, the proportion of correctly mapped reads) between two methods was tested using the chi-square test. In the case of the real data it may be more appropriate to treat samples as the statistical unit because there may be differences between samples that affect the performance of different methods (e.g. different levels of mismatch with the reference genes). Therefore, we also used the Wilcoxon signed-rank test to perform a paired comparison of the median proportions of reads from the biological samples mapped by each method. The significance threshold for both tests was set at 0.01.

## RESULTS

### Accurate CDR3 extraction and VDJ identification

**Figure 4.**
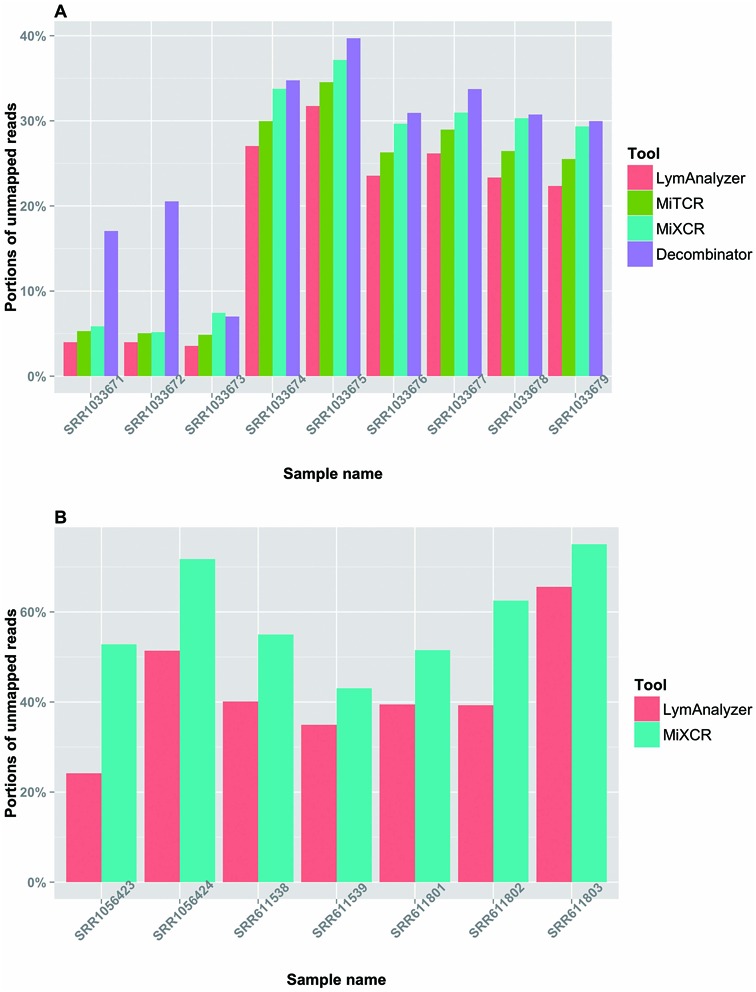
Results based on real data set. (**A**) The comparison of mapping completeness of TCR data among LymAnalyzer, MiTCR, MiXCR and Decombinator. LymAnalyzer outperformed the other tools in all nine sample based on the completeness of the alignment. (**B**) The comparison of mapping completeness of IG data between LymAnalyzer and MiXCR. LymAnalyzer consistently mapped larger proportions of reads compared to MiXCR in all the samples.

LymAnalyzer was first applied to a data set in the public domain (SRA: PRJNA229070), consisting of short read TCR sequences from nine samples. LymAnalyzer consistently mapped a significantly higher proportion of the reads (Figure 4A), compared to MiTCR, MiXCR and Decombinator (*P* < 0.01 in all cases; See Materials and Methods for details of statistical tests). The decline in the proportion of extracted reads from all three tools from sample SRR103674 to sample SRR1033679 was due to differences in sequencing quality. We also compared the performance of LymAnalyzer with MiXCR on IG sequences using a publicly available data set (SRA: SRP017087). Because many of the reads in this data set do not cover the CDR3 region, a large proportion of the reads remained unmapped by both tools; however, LymAnalyzer mapped a larger portion of reads compared to MiXCR in all cases (*P* < 0.01).

**Figure 5.**
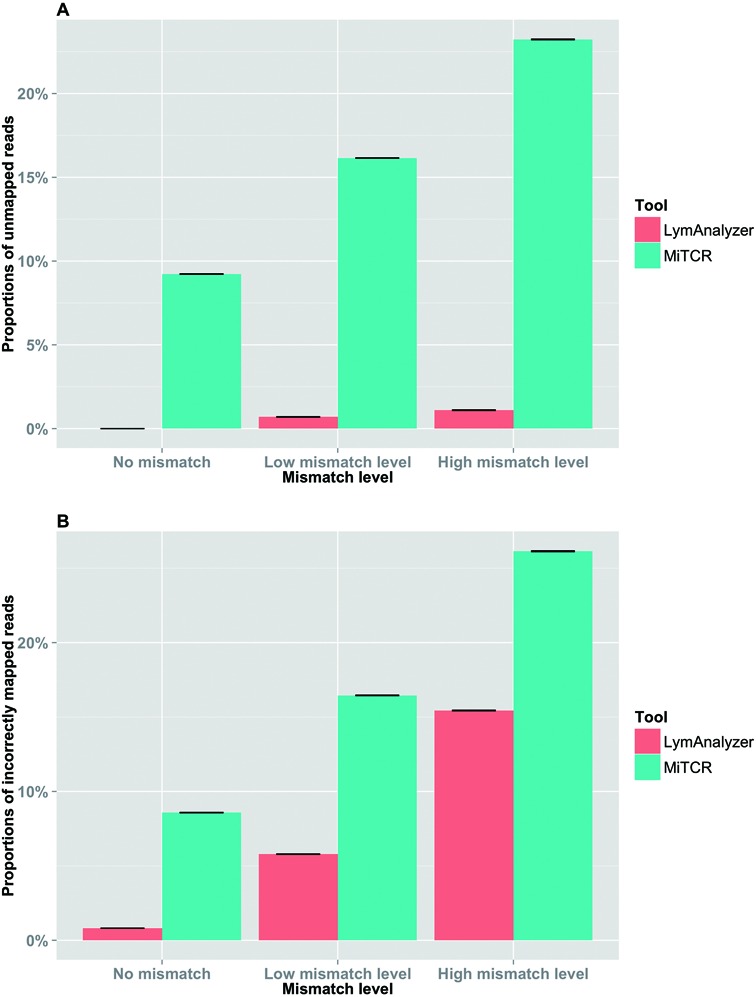
Results based on simulated TCR data on the allele name level. (**A**) Comparison of completeness of the results from LymAnalyzer and MiTCR. (**B**) Comparison of the accuracy of LymAnalyzer and MiTCR. The completeness and accuracy values shown are the means of the results from twenty simulated samples. The error bars shown at the top of each bar indicate the standard error of the mean of the simulated data sets.

**Figure 6.**
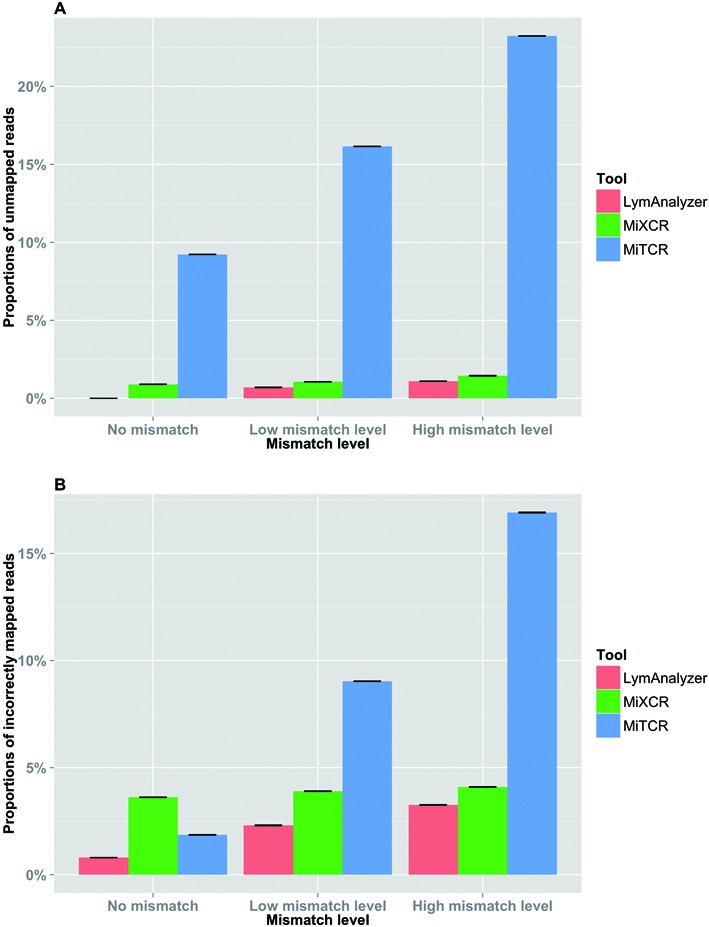
Results based on simulated TCR data at the gene name level. (**A**) Comparison of the completeness of the results from LymAnalyzer, MiTCR and MiXCR. LymAnalyzer had the highest completeness among the three tools at all mismatch levels. The completeness decreased with increasing mismatch level; however LymAnalyzer retained above 98% completeness even at the highest mismatch level. (**B**) Comparison of the accuracy of LymAnalyzer, MiTCR and MiXCR at different mismatch levels; LymAnalyzer outperformed MiTCR and MiXCR in terms of accuracy.

**Figure 7.**
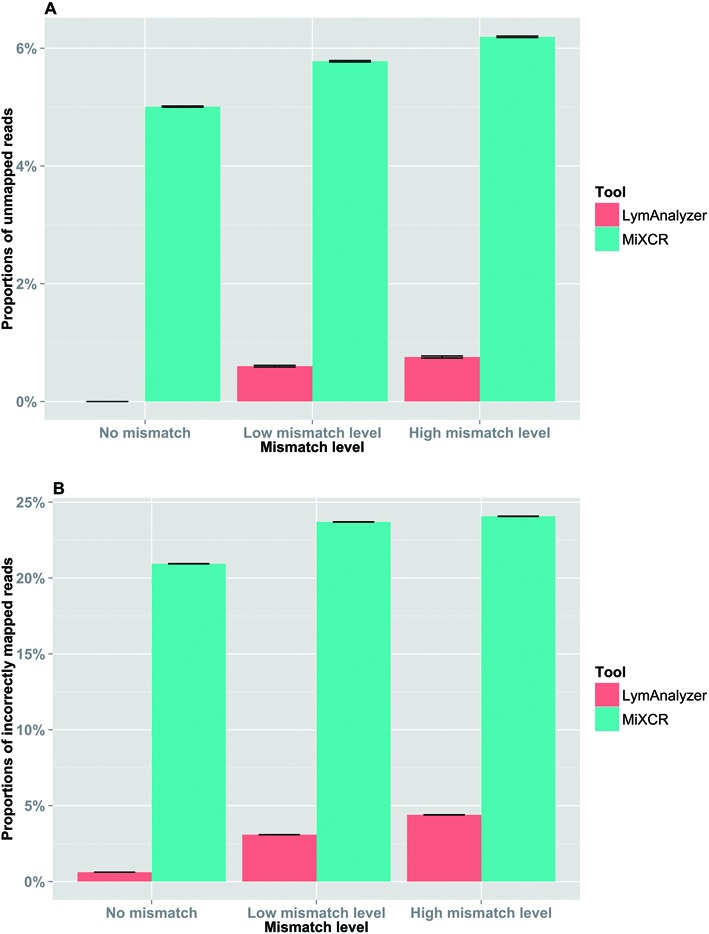
Results based on simulated IG data at the gene name level. (**A**) The comparison of completeness of the results from LymAnalyzer and MiXCR. (**B**) The comparison of the accuracy of LymAnalyzer and MiXCR. LymAnalyzer had both significantly improved accuracy and completeness compared to MiXCR.

We used simulated data sets to investigate the accuracy and completeness of the results generated by LymAnalyzer. Each simulation consisted of VDJ recombination together with different mismatch levels. For simulated TCR data, in the absence of mismatches, LymAnalyzer can map all of the sequences, whereas MiTCR only mapped 91% of the sequences (Figure 5A). As the mismatch level increased, the number of reads that MiTCR and LymAnalyzer mapped declined gradually, as expected; however, LymAnalyzer still mapped a greater proportion of the reads than MiTCR. In addition to mapping a greater proportion of the reads, LymAnalyzer was also significantly more accurate than other methods, with 99.25% of the sequences mapped correctly, compared to 91.45% correctly mapped sequences with MiTCR in the absence of mismatches (Figure 5B). We also compared the results from Decombinator and MiXCR based on simulated data (Supplementary Figure S1). However, Decombinator and MiXCR can only give us the particular gene name of each sequence, instead of allele name, which is the standard output of MiTCR and LymAnalyzer. Therefore we only compared the results under gene name level. Under the high mutation level, Decombinator missed more than one third of the sequences. Therefore we compared the performance of LymAnalyzer, MiTCR and MiXCR separately (Figure 6). At the gene name level, both LymAnalyzer and MiTCR had increased accuracy as expected. MiTCR had higher accuracy (98.1%) comparing to MiXCR (96.4%) in the no mismatch data set. However, as the mismatch level increased, MiXCR achieved higher accuracy than MiTCR. LymAnalyzer still achieved the highest accuracy and completeness among the three tools at all mismatch levels. For simulated IG data, we compared LymAnalyzer with MiXCR since they are, to date, the only tools that can process IG NGS data (Figure 7). LymAnalyzer showed both improved accuracy and completeness relative to MiXCR. In terms of accuracy, in the absence of mismatches, MiXCR mapped more than 20% of the sequences incorrectly, compared to 0.7% mapping errors in LymAnalzyer. Under the high mismatch level, LymAnalyzer retained accuracy above 95%, while the accuracy of MiXCR declined to 75.9%.

### Running time

LymAnalyzer runs on Windows, Linux and Mac OS X. We tested the running performance of LymAnalyzer on both a Linux cluster and a personal computer (MacBook). On a MacBook, for 125 000–200 000 sequences, the full analysis can be finished in 9–12 s. For large data sets, with 10–15 million sequences, the full analysis can be accomplished in 25–40 min on our cluster server (Hardware configuration: 6-core 2.2Ghz AMD Opteron Processor 2427 with 32 GB memory). As can be seen from the plot in Supplementary Figure S2, the running time scales linearly with the number of reads. The estimated processing speed of LymAnalyzer for sequences of 100 bp long is 8461 reads per second (Table 1).

**Table 1.** Feature comparisons of different TCR/IG sequencing analysis tools

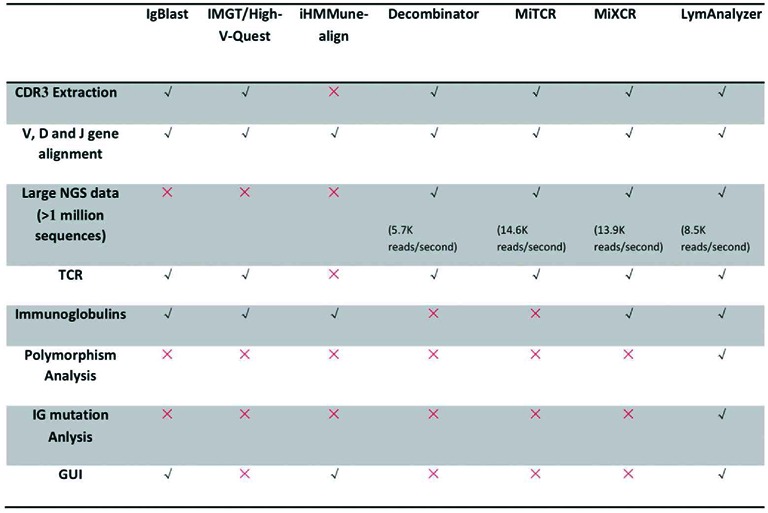							

### Additional features of LymAnalyzer

In addition to features that are common to existing tools, LymAnalyzer can perform polymorphism analysis and generate hypermutation trees for IG sequences. LymAnalyzer provides both command line and GUI version and is implemented in JAVA for cross-platform application. A comparison of features available in different tools is provided in Table 1.

In order to test if our SNP calling algorithm is capable of recognizing potential unreported alleles, we manually modified the reference gene database, retaining just one allele of each distinct V gene. We then used LymAnalyzer to map a subset of 125 000 reads from sample SRR1033674 to this modified reference gene database. From the result file, we found seven putative SNPs (Table 2). By mapping these suspected SNPs back to the original reference gene database, we found that all but one of them can be accounted for by the known alleles that were removed from our reference gene database at the beginning of the test (Table 2). There was one variant of TRBV29–1*03, corresponding to a substitution from A to C, that could not be mapped to an existing allele in the IMGT database. Given the high frequency (45.44%) and read count (4488) for this mutation, it could correspond to an allele that is not found in the database. In support of this hypothesis we found that there is a known A/C SNP (rs17214) at the genomic position corresponding to this mutation in dbSNP (15). This variant leads to an amino acid change (Methionine to Leucine) on TRVB29–1.

**Table 2.** Suspected SNPs and their true allele on the V genes

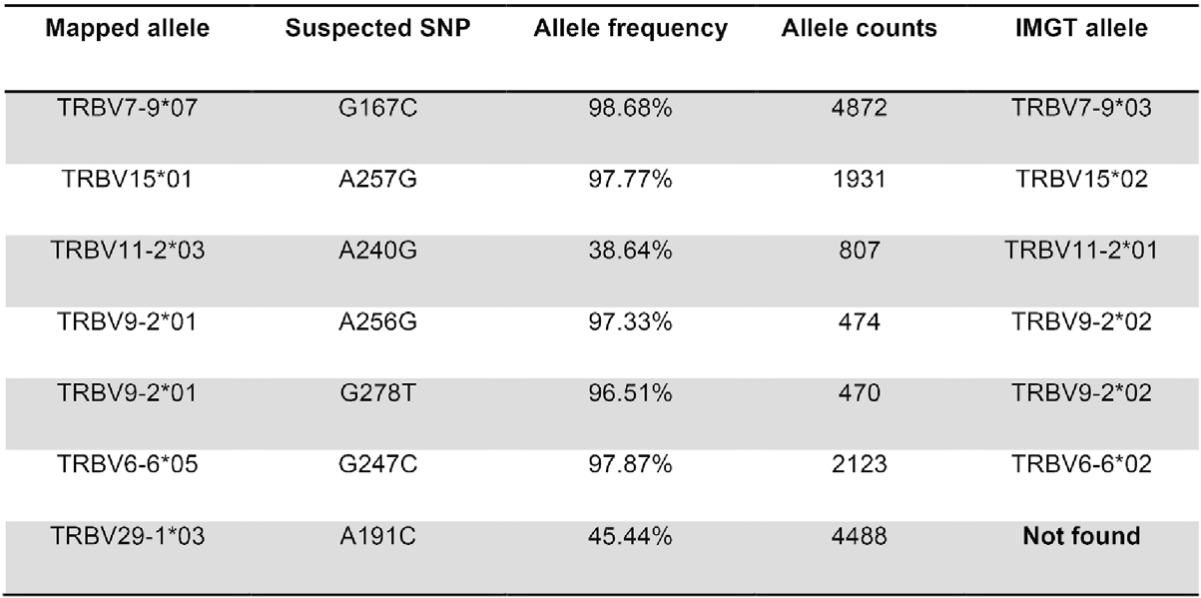				

**Figure 8.**
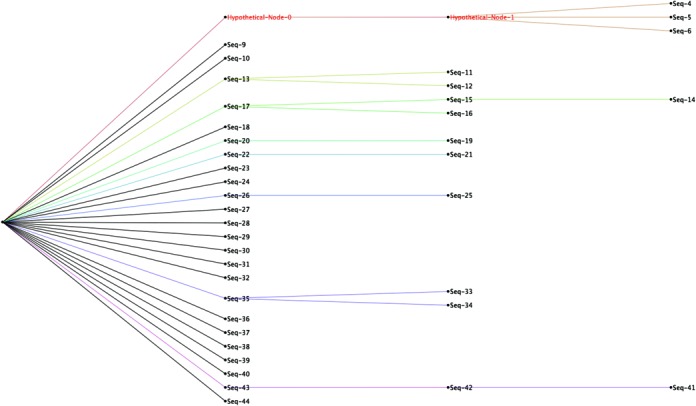
Example of a mutation tree generated by LymAnalyzer. The tree is visualized using FigTree (http://tree.bio.ed.ac.uk/software/figtree/). Each node represents an individual clone. The nodes on the same level are one Levenshtein step (nucleotide change) away from their corresponding nodes on the previous and subsequent layers. The hypothetical nodes are shown in red. These are required to connect the nodes that are more than one step away from the closest observed sequence.

Mutation trees are generated in Newick format and can be visualized using several existing software packages (Figure 8). The tree does not necessarily represent the real mutation process that took place; it shows the minimal steps that can explain the observed sequences. Adjacent layers are separated by a Levenshtein distance of one, which represents one nucleotide change. Each of the nodes in a given layer is one step away from the nodes to which it is linked in the previous and subsequent layers. However it is not guaranteed that there is always a parent node that is one Levenshtein distance away from the current node. Therefore we create a hypothetical node in each layer of the tree. The hypothetical node is not a real sequence that exists in the data set, but instead represents the collection of unobserved intermediate sequences between two nodes that are separated by a Levenshtein distance greater than one.

## DISCUSSION

Next generation sequencing technology gives researchers an opportunity to study lymphocyte repertoire diversity at high resolution. However current bioinformatics pipelines for identification and annotation of large TCR/IG sequence data sets are unsatisfactory due to their suboptimal accuracy and completeness. Here we present LymAnalyzer, a software package for comprehensive analysis of TCR/IG sequence data.

LymAnalyzer consists of four functional components: VDJ gene identification followed by CDR3 extraction, SNP calling and lineage mutation tree generation. We performed multiple tests of accuracy using publicly available and simulated data sets and compared the performance of LymAnalyzer to existing tools (MiTCR, MiXCR and Decombinator). In our evaluation using real data, LymAnalyzer mapped more reads than the other tools. In terms of accuracy, we have shown using simulated data that LymAnalyzer provides significantly improved mapping accuracy compared to MiTCR, MiXCR and Decombinator. MiTCR had the fastest running performance among the tools; however, it trades accuracy and completeness for speed. Given that TCR/IG sequencing data sets are tractable on a personal computer for typical data sets or on computer clusters for large projects this trade off is unnecessary. Despite significant improvement in accuracy and completeness, the running time of LymAnalyzer is better than Decombinator and remains comparable to MiTCR and MiXCR.

The majority of lymphocyte sequence analysis tools can only process TCRs. LymAnalyzer makes the analysis of IGs also available. Furthermore, LymAnalyzer is to date the only tool that can generate mutation trees for IGs. Another novel feature of LymAnalyzer is the ability to detect the SNPs. We tested the reliability of this function by running LymAnalyzer on the same data set again with the reference gene database that only kept one representative allele for each gene and compared the results from both runs. Indeed, LymAnalyzer revealed the SNPs, which can also be found in the removed alleles. However, the accuracy of the SNP detection can be hampered by allelic imbalance. We only report the suspected SNPs that exceed particular mutation rate threshold (10%), and we may miss some imbalanced alleles that are lower than this threshold. Therefore, we set this threshold as an adjustable parameter. Users can change this threshold value based on their requirements.

Previous studies have shown that the IMGT database appears to be incomplete, as many reported IG heavy chain variable alleles are not found in the database (16–21).Moreover, many IG heavy chain variable alleles polymorphisms may have been reported in error (22). The unreported SNP on TRBV29–1 found in our study shows that there are also TCR beta chain variable alleles missing from the IMGT database. A more updated and robust reference gene database for TCR/IG sequences is required. By taking advantage of the increased availability of TCR/IG sequence data sets, the SNP detection function implemented in LymAnalyzer could help to discover novel alleles and improve the coverage of the TCR/IG reference gene database.

## Supplementary Material

SUPPLEMENTARY DATA
