# Supplementary material for: LymAnalyzer: a tool for comprehensive analysis of next generation sequencing data of T cell receptors and immunoglobulins
Source: Nucleic Acids Res. 2015 Oct 7;44(4):e31. doi: 10.1093/nar/gkv1016 (PMC4770197; doi:10.1093/nar/gkv1016)
Supplement: SUPPLEMENTARY DATA [file supp_44_4_e31__index.html]

LymAnalyzer: a tool for comprehensive analysis of next generation sequencing data of T cell receptors and immunoglobulins — LymAnalyzer: a tool for comprehensive analysis of next generation sequencing data of T cell receptors and immunoglobulins — SUPPLEMENTARY DATA 

# LymAnalyzer: a tool for comprehensive analysis of next generation sequencing data of T cell receptors and immunoglobulins

## SUPPLEMENTARY DATA

- SUPPLEMENTARY DATA
