# Supplementary material for: LymAnalyzer: a tool for comprehensive analysis of next generation sequencing data of T cell receptors and immunoglobulins
Source: Nucleic Acids Res. 2015 Oct 7;44(4):e31. doi: 10.1093/nar/gkv1016 (PMC4770197; doi:10.1093/nar/gkv1016)
Supplement: SUPPLEMENTARY DATA [file supp_gkv1016_nar-01693-met-g-2015-File013.pdf]

Supplementary Figure Legends

Supplementary figure 1. Comparisons of completeness and accuracy among LymAnalyzer, MiXCR and Decombinator based on simulated TCR data on the gene name level.

Supplementary figure 2. Running performance of LymAnalyzer.
